# Risk Factors for Dysfunctional Liver Graft Response: A Case–Control Study

**DOI:** 10.1111/nhs.70357

**Published:** 2026-05-17

**Authors:** Layana de Paula Cavalcante, João Cruz Neto, Maria Beatriz Freire Alves, Francisca Alessandra Peixoto da Cunha, Ana Clara de Brito Gomes, Reinaldo Gutierrez Barreiro, Viviane Martins da Silva, Marcos Venícios de Oliveira Lopes

**Affiliations:** ^1^ Federal University of Ceará Fortaleza Brazil; ^2^ Universidad Surcolombiana Huila Colombia

**Keywords:** clinical decision‐making, liver transplantation, nursing diagnosis, primary graft dysfunction, risk factors

## Abstract

The purpose of this article was to identify recipient‐, donor‐, and graft‐related risk factors associated with dysfunctional liver graft response following liver transplantation. A case–control study was developed based on medical records of adult liver transplant recipients were analyzed. Cases were defined by the occurrence of dysfunctional liver graft response within the first seven postoperative days or by death/retransplantation within 6 months. Controls had preserved graft function and survived without retransplantation. Logistic regression analysis was performed to identify associated risk factors. Recipient‐related risk factors included younger age, renal dysfunction, higher body mass index, and reduced blood transfusion support. Donor‐related factors included older age, male sex, and elevated serum sodium levels. Prolonged warm and cold ischemia times were also associated with dysfunction. These findings supported the development of the proposed nursing diagnosis, “Risk for Dysfunctional Liver Graft Response.” The dysfunctional liver graft response is a multifactorial phenomenon involving recipient, donor, and graft‐related factors. These findings contribute to highlight the relevance of nursing involvement throughout transplantation pathways.

## Key Points

- This study addressed the challenge of early identification of risk factors for dysfunctional liver graft response, a critical determinant of patient outcomes following liver transplantation.
- Nine independent variables were identified as significant predictors of graft dysfunction, encompassing recipient (age, BMI, renal function, transfusion volume), donor (age, sex, sodium levels), and graft‐related (ischemia times) factors.
- Findings are applicable to adult liver transplant recipients and transplant care teams, especially nurses involved in immediate postoperative care in intensive care units and transplant wards. The research supports clinical protocols and the integration of a novel nursing diagnosis into care systems.

**Abbreviations**
ALT: alanine aminotransferase
AST: aspartate aminotransferase
BMI: body mass index
CI: confidence interval
DLGR: dysfunctional liver graft response
EAD: early allograft dysfunction
IBSA: intraoperative blood salvage autologous transfusion
ICU: intensive care unit
INR: international normalized ratio
MELD: model for end‐stage liver disease
NANDA‐I: NANDA International
OR: odds ratio
RDLGR: risk for dysfunctional liver graft response
SD: standard deviation
STROBE: strengthening the reporting of observational studies in epidemiology
U: Mann–Whitney U statistic

## 1 Introduction

Liver transplantation is indicated for a range of hepatic conditions that can be grouped into five major categories: cholestatic diseases, hepatocellular failure, metabolic diseases, vascular diseases, and neoplastic conditions (Pietrobattista et al. 2025). The assessment of these conditions includes evaluation of disease severity, whether acute or chronic, active or inactive, and whether the condition is mild, moderate, or severe. In general, serum aminotransferase levels are useful noninvasive markers of disease activity (Kamath et al. 2001).

The severity of liver disease is primarily assessed using two prognostic models: the Model for End‐Stage Liver Disease (MELD) and the Child‐Pugh score. MELD is based on three laboratory parameters: serum creatinine, bilirubin, and the International Normalized Ratio (INR). A MELD score of ≥ 15 indicates severe liver dysfunction and priority for transplantation (Hudson et al. 2025). The Child‐Pugh classification combines three laboratory parameters with two clinical findings (ascites and hepatic encephalopathy), with Class C (≥ 10 points) representing the most severe clinical condition (Wang et al. 2026).

Absolute contraindications to liver transplantation include recent use of alcohol or illicit drugs (within 6 months), advanced cardiopulmonary disease, inability to adhere to immunosuppressive regimens, extrahepatic malignancies, uncontrolled sepsis, and evidence of irreversible severe neurological disorders (Jothimani et al. 2026). In addition, relative contraindications include advanced age, prior abdominal surgeries, severe malnutrition, or obesity, renal dysfunction (creatinine > 2 mg/dL), hepatobiliary tumors, portal vein thrombosis, and HIV infection (Busuttil and Klintmalm 2015).

Postoperatively, liver transplant recipients are routinely admitted to intensive care units (ICUs), where they receive multidisciplinary care focused on optimizing immunosuppressive therapy, monitoring hepatic, renal, hematological, and biochemical function, and early detection of infections. In addition, emotional, social, and economic factors are evaluated to facilitate adaptation to postdischarge life (Xu et al. 2025).

In this context, the role of nurses is essential throughout the transplantation process. Their responsibilities span from pretransplant evaluation of recipients, identification and maintenance of potential donors (including those with suspected brain death), family interviews to obtain consent for organ donation, coordination and preparation for organ procurement surgery, intraoperative participation, and postoperative care in both immediate ICU settings and long‐term outpatient follow‐up (Karataş and Balas 2025). Such evaluation enhances care delivery throughout all stages of the donation and transplantation process (López‐Montesinos et al. 2010; Akbulut et al. 2022).

In this regard, the NANDA International (NANDA‐I) taxonomy provides a standardized framework for documenting clinical nursing practice and supporting professional communication. Importantly, it also fosters the advancement of nursing as an independent discipline grounded in scientific knowledge by defining nursing diagnoses as human responses that are sensitive to nursing interventions (Herdman et al. 2024).

The proposed nursing diagnosis, Risk for dysfunctional liver graft response (RDLGR), aims to expand and update the catalog of nursing diagnoses, particularly addressing gaps in transplant‐related care. The study seeks to guide clinical decision‐making, improve patient outcomes, and support nursing practice within the context of liver transplantation, where the literature on nursing diagnoses remains limited. Given that all liver transplant recipients are inherently at high risk for some degree of graft dysfunction (Al‐Freah et al. 2017), the ability of nurses to detect early signs of graft failure is critical.

Liver graft impairment in the early postoperative period has been described using different terms in the literature, including early allograft dysfunction (EAD), primary graft dysfunction, and primary nonfunction. Although related, these concepts are not interchangeable. EAD generally refers to early biochemical and clinical evidence of impaired graft function, whereas primary non‐function describes severe and irreversible graft failure, usually leading to urgent retransplantation or death. In the present study, we adopted the term dysfunctional liver graft response (DLGR) as the main outcome of interest, because it better reflects the clinical phenomenon underlying the proposed nursing diagnosis while encompassing early graft impairment identified through predefined postoperative criteria. Operationally, case classification was based on the occurrence of graft dysfunction within the first seven postoperative days, according to the criteria proposed by Olthoff et al. (2010), and/or death or retransplantation within 6 months after surgery. Therefore, DLGR is used consistently throughout this manuscript as the primary outcome term, while EAD and primary non‐function are mentioned only when referring to established concepts in the previous literature. Thus, this study aims to analyze the associations between etiological factors and the development of dysfunctional graft responses in liver transplant recipients.

## 2 Methods

### 2.1 Study Design and Sampling Framework

This was an individual unmatched case–control study, in which the medical record of each liver transplant recipient served as the unit of analysis. The research was conducted using secondary data obtained from patient medical records. This manuscript was prepared in accordance with the Strengthening the Reporting of Observational Studies in Epidemiology (STROBE) checklist, specifically the version for case–control studies (von Elm et al. 2008).

Data collection was carried out at a public hospital located in Northeast Brazil, specifically within the Medical Records and Statistics Service. This hospital is internationally recognized for its liver transplant program, with outcomes surpassing those of Mexico, Chile, and other South American countries. The multidisciplinary transplant team includes physicians, nurses, physical therapists, psychologists, nutritionists, social workers, and occupational therapists, ensuring comprehensive patient care.

Sample size was estimated for an unmatched case–control design, considering a 95% confidence level, 80% statistical power, a case‐to‐control ratio of 2:1, and a conservative exposure frequency of 50% among controls. An odds ratio of 1.6 was adopted as the minimum clinically relevant effect size. Based on these parameters, the minimum required sample was estimated at 651 medical records, corresponding to 434 cases and 217 controls. However, after applying the eligibility criteria and excluding records with incomplete or inconsistent data, 588 records were included in the final analysis, comprising 371 cases and 217 controls. As a result, the estimated statistical power decreased from 80% to 73%, which may have reduced the study's ability to detect weaker associations.

The study population included adult patients (≥ 18 years old), of both sexes, who underwent liver transplantation with follow‐up data available for the first seven postoperative days. Medical records were included if complete and consistent during this period. Exclusion criteria were combined transplant procedures and records with missing or inconsistent data.

The study population included adult patients (≥ 18 years old), of both sexes, who underwent liver transplantation and had follow‐up data available for the first seven postoperative days. Medical records were considered eligible when they contained complete and consistent information for the variables of interest. Records related to combined transplantation procedures or with missing/inconsistent essential data were excluded. Cases were defined as patients who developed dysfunctional liver graft response, operationalized according to the criteria proposed by Olthoff et al. (2010), including total bilirubin ≥ 10 mg/dL, INR ≥ 1.6, and/or AST or ALT > 2000 IU/L within the first seven postoperative days, or death or retransplantation within 6 months after surgery. Controls were defined as patients with preserved graft function and hemodynamic stability during the first seven postoperative days, with no retransplantation and no death within 6 months after transplantation.

This study did not involve random allocation, as it was a retrospective observational case–control study based on secondary data from medical records. Participants were not assigned to treatment groups; rather, they were classified after transplantation as cases or controls according to the occurrence of dysfunctional liver graft response and follow‐up outcomes. Both groups were drawn from the same hospital‐based source population and were selected using predefined eligibility criteria.

### 2.2 Data Collection Tools and Methods

Data were collected using a structured form designed to gather demographic, clinical, and risk factor information relevant to the proposed nursing diagnosis RDRLG. The instrument was developed on the basis of etiological factors identified in a previous phase of the study, for which operational definitions had been constructed.

Donor‐related variables included: sex, age, blood type, living or deceased status, cause of brain death, weight, height, skin color, presence of comorbidities, hemodynamic data, laboratory tests, surgical times, graft perfusion times, presence of steatosis, and anatomical variations. Recipient‐related variables included: sex, age, blood type, primary diagnosis, presence of ascites, hepatic encephalopathy, alcohol use, smoking status, comorbidities, blood transfusion requirement, MELD score, and Child‐Pugh score.

These variables are routinely recorded in standardized forms used by liver transplant services. The instrument was previously evaluated in a pilot study to ensure clarity and applicability. Data were collected by the lead researcher between December 2021 and April 2022 from physical medical records stored in the hospital's Medical Records and Statistics Service (SAME). A list of patients who had undergone liver transplantation was obtained, and the corresponding records were retrieved for review. Data collection focused on the first seven postoperative days and included information on retransplantation or death within 6 months after the procedure.

### 2.3 Data Analysis

Data were entered into a Microsoft Excel spreadsheet and analyzed using R software, version 4.0.3. Descriptive analysis included absolute and relative frequencies for categorical variables and measures of central tendency and dispersion for continuous variables. The distribution of continuous variables was assessed using the Shapiro–Wilk test.

For bivariate analysis, Pearson's chi‐squared test was used to assess associations between categorical variables, and Fisher's exact test was applied when expected cell frequencies were less than five. For continuous variables, Student's *t*‐test was planned for variables with a normal distribution, whereas the Mann–Whitney U test was used for variables with a nonnormal distribution. Crude odds ratios and their respective 95% confidence intervals were calculated for categorical predictors.

To identify factors independently associated with dysfunctional liver graft response, univariate and multivariate logistic regression analyzes were performed, considering the binary nature of the outcome and the case–control design. Variables with *p* < 0.20 in the univariate analysis were considered eligible for inclusion in the hierarchical multivariate model. Recipient‐related variables were entered at the first level, donor‐related variables at the second level, and graft‐related variables at the third level. Adjusted odds ratios with 95% confidence intervals were estimated. Model performance and fit were assessed using the Wald test, Omnibus test, and Hosmer–Lemeshow goodness‐of‐fit test. Statistical significance was set at *p* < 0.05.

Because the primary objective of this study was to identify variables associated with dysfunctional liver graft response in a retrospective case–control design, multivariable logistic regression was selected as the main analytical strategy. This approach was considered appropriate for the binary outcome and for the simultaneous assessment of multiple recipient‐, donor‐, and graft‐related characteristics. The study was not originally designed to estimate causal effects for a single exposure under a formal causal inference framework.

### 2.4 Ethical and Institutional Approvals

This study was approved by the institution's Research Ethics Committee under protocol number 5.208.291. Because this was a retrospective study based on secondary data obtained from medical records, all procedures were conducted in accordance with applicable ethical standards for research involving human data and with institutional requirements for confidentiality and data protection.

## 3 Results

This study evaluated 588 liver transplant recipients. Of these, 371 (63.1%) met the criteria for dysfunctional liver graft response (DLGR) and were classified as cases, whereas 217 (36.9%) did not meet the diagnostic criteria for graft dysfunction and were classified as controls. Most recipients were male (63.4%), with a mean age of 52.7 years (SD = 11.4). Most patients were of mixed race (91%), and Child‐Pugh class B was the most frequent classification in both groups (Table 1).

**TABLE 1:** Comparison of the clinical characteristics of liver transplant recipients.

Variables	Case (*n* = 371)	Control (*n* = 217)	*χ* ^2^	p	OR	95% CI
	*n* (%)	*n* (%)				
Sex						
Female	128 (34.5)	67 (30.9)	0.81^a^	0.368	1.18	0.82–1.69
Male	243 (65.5)	150 (69.1)				
Race						
Nonbrown	33 (8.9)	17 (7.8)	0.08^a^	0.770	1.15	0.60–2.26
Brown	338 (91.1)	200 (92.2)				
Childpugh						
A < 6 points	61 (16.4)	32 (14.7)	5.03^a^	0.081	—	—
B < 7–9 points	200 (53.9)	102 (47.0)				
C < 10–15 points	108 (291)	83 (38.3)				
Arterial hypertension						
Yes	71 (19.1)	47 (21.7)	0.54^a^	0.461	0.86	0.57–1.29
No	300 (80.9)	170 (78.3)				
Diabetes mellitus						
Yes	82 (22.1)	58 (26.7)	1.61^a^	0.204	0.78	0.53–1.15
No	289 (77.9)	159 (73.3)				
Encephalopathy						
Yes	141 (38.0)	104 (47.9)	5.54^a^	0.019	0.67	0.47–0.93
No	230 (62.0)	113 (52.1)				
Alcoholism						
Yes	191 (51.5)	129 (59.5)	3.50^a^	0.061	0.72	0.52–1.02
No	180 (48.5)	88 (40.5)				
Smoking						
Yes	93 (25.1)	63 (29.0)	1.10^a^	0.293	0.82	0.56–1.19
No	278 (74.9)	154 (71.0)				
Renal dysfunction						
Yes	74 (19.9)	24 (11.1)	7.79^a^	0.005	2.00	1.22–3.29
No	297 (80.1)	193 (88.9)				

Blood type	Rh (Case)		Rh (Control)					
	neg	pos	neg	pos				
A	6 (1.6)	101 (27.2)	7 (1.8)	54 (14.5)	0.94^b^	0.794	—	—
AB	1 (0.2)	13 (3.5)	1 (0.4)	9 (4.1)				
B	5 (1.3)	43 (11.6)	1 (0.4)	19 (8.8)				
O	9 (2.4)	136 (36.7)	4 (1.8)	86 (39.6)				

Variables	Mean rank (Case)	Mean rank (Control)	U	*p*
Age	282.7	314.6	35 886.5	0.028^c^
No. of transfusions	283.3	313.6	36 106.5	0.017^c^
MELD	285.4	310.0	36 895.5	0.087^c^
Weight	300.2	284.8	38 140.0	0.288^c^
Height	291.4	295.6	39 281.0	0.772^c^
BMI	298.4	285.2	38 243.5	0.364^c^
Creatinine × 300%	289.6	302.8	38 455.0	0.365^c^

^a^ Pearson's chi‐squared.

^b^ Mantel–Haenszel chi‐squared.

^c^ Mann–Whitney Test; neg: Negative Rh; pos: Positive Rh; U: Mann–Whitney statistics; *p* < 0.05 was considered statistically significant.

Comparison of recipients' clinical characteristics showed no statistically significant association between biological sex and DLGR, although male sex predominated in both groups. Similarly, mixed race was the most frequent category among recipients, with no significant association with the outcome. The severity of liver disease, assessed using Child‐Pugh score, was similarly distributed between groups, with most patients classified as class B. Arterial hypertension and diabetes mellitus were also not significantly associated with DLGR, suggesting that although clinically relevant, these comorbidities were not independent risk factors in this sample.

Hepatic encephalopathy was significantly less frequent among individuals who developed DLGR (*p* = 0.019; OR = 0.67; 95% CI: 0.47–0.93), suggesting either a possible protective association or more selective transplantation of patients with encephalopathy. Although alcoholism was more frequent in the control group, the difference did not reach statistical significance, despite showing a trend toward association. Smoking status and blood type were not associated with DLGR. The only categorical recipient‐related clinical factor significantly associated with the outcome was pretransplant renal dysfunction (*p* = 0.005; OR = 2.00; 95% CI: 1.22–3.29), which was more frequent in the DLGR group and doubled the likelihood of dysfunction.

Regarding donors, there was also a predominance of males, representing approximately two‐thirds of the cases, and their mean age was higher than that of recipients (Table 2). Among quantitative variables, recipients who developed DLGR were significantly younger (*p* = 0.028), suggesting a higher risk among younger transplant recipients. These individuals also received significantly fewer blood transfusions (*p* = 0.017), which may be related to reduced intraoperative or postoperative hemodynamic support. No statistically significant differences were observed between groups for MELD scores, body weight, height, body mass index, or creatinine levels, indicating homogeneity across these parameters.

**TABLE 2:** Comparison of the clinical characteristics of liver transplant donors.

Variables	Case (*n* = 371)		Control (*n* = 217)		*χ* ^2^	*p*	OR	95% CI
	*n* (%)		*n* (%)					
Sex								
Female	107 (29.2)		76 (35.3)		2.13^a^	0.144	0.75	0.52–1.08
Male	260 (70.8)		139 (64.7)					
Race								
Nonbrown	312 (84.5)		178 (82.0)		0.46^a^	0.495	1.20	0.76–1.87
Brown	57 (15.5)		39 (18.0)					
Blood compatibility								
Yes	2 (0.5)		1 (0.5)		—	1.000^b^	1.17	0.10–13.0
No	366 (99.5)		214 (99.5)					

Blood type	Rh (Case)		Rh (Control)					
	neg	pos	neg	pos				
A	6 (1.9)	101 (32.2)	7 (3.9)	54 (29.8)	0.02^c^	0.892	—	—
AB	1 (0.3)	13 (4.1)	1 (0.5)	9 (5.0)				
B	5 (1.6)	43 (13.7)	1 (0.5)	19 (10.5)				
O	9 (2.9)	136 (43.3)	4 (2.2)	86 (47.5)				

Variables	Mean rank (Case)	Mean rank (Control)	U	*p*
Age	310.7	261.5	33 056.5	0.001^d^
Weight	305.2	272.2	35 422.5	0.022^d^
Height	303.4	275.3	36 088.5	0.050^d^
BMI	304.1	274.1	35 824.0	0.037^d^
Urea	292.5	272.3	35 089.0	0.157^d^
Creatinine	282.7	290.3	36 857.5	0.592^d^
Sodium	300.0	259.4	32 321.0	0.005^d^
Aspartate Aminotransferase	288.5	267.0	33 854.5	0.127^d^
Alanine aminotransferase	291.9	261.2	32 585.5	0.030^d^

^a^ Pearson chi‐squared.

^b^ Fisher exact test.

^c^ Mantel–Haenszel chi‐squared.

^d^ Mann–Whitney test; neg: Negative Rh; pos: Positive Rh; U: Mann–Whitney statistics; *p* < 0.05 was considered statistically significant.

Analysis of donor clinical data showed no significant association between donor sex, skin color, or blood type compatibility and DLGR in recipients. However, several relevant findings emerged. Donor age was significantly higher in cases with DLGR (*p* = 0.001). In addition, donors whose grafts were associated with DLGR had, on average, higher body weight (*p* = 0.022), higher body mass index (*p* = 0.037), and higher serum sodium levels (*p* = 0.005). These findings suggest that donor physiological and metabolic characteristics play an important role in graft viability and recipient adaptation.

Other donor laboratory parameters, such as urea, creatinine, transaminases (AST and ALT), and times related to organ procurement and hepatectomy, were not significantly associated with graft dysfunction. Ischemia times were also clinically relevant. Both warm ischemia time (*p* = 0.001) and cold ischemia time (*p* = 0.022) were significantly longer in transplants that resulted in DLGR, underscoring the importance of strict intraoperative time management for graft preservation.

Most grafts originated locally, and hepatic steatosis under 30% was observed in approximately 80% of transplants (Table 3). Surgical and graft characteristics showed a similar pattern in both groups, with no significant association with outcome. Although most cases involved mild steatosis (< 30%), steatosis between 30% and 60% showed a tendency toward lower risk of DLGR, warranting further investigation.

**TABLE 3:** Comparison of graft/surgical liver characteristics.

Variables	Case (*n* = 371)	Control (*n* = 217)	*χ* ^2^	*p*	OR	95% CI
	*n* (%)	*n* (%)				
Graft origin						
Local	257 (69.3)	167 (77.0)	5.09^a^	0.078	—	—
National	74 (19.9)	28 (12.9)				
Regional	40 (10.8)	22 (10.1)				
Degree of steatosis						
< 30%	305 (82.4)	192 (88.5)	3.40^a^	0.065	0.61	0.37–1.00
30% to 60%	65 (17.6)	25 (11.5)				

Variables	Mean rank (Case)	Mean rank (Control)	U	*p*
Warm ischemia time	298.1	251.5	30 492.0	0.001^b^
Cold ischemia time	306.8	273.5	35 697.5	0.022^b^
Extraction time	294.1	294.7	40 166.5	0.965^b^
Start of hepatectomy	299.1	291.8	39 255.0	0.614^b^
End of hepatectomy	299.1	291.8	39 262.0	0.616^b^
Total surgery time	283.8	300.7	37 933.0	0.243^b^

^a^ Pearson's chi‐squared.

^b^ Mann–Whitney test; U: Mann–Whitney statistics; *p* < 0.05 was considered statistically significant.

Multivariate logistic regression analysis identified nine independent variables associated with the occurrence of DLGR (Table 4). Among recipient variables, younger age was associated with increased risk, with an estimated 2% reduction in risk per additional year of age (*p* = 0.017; OR = 0.98; 95% CI: 0.97–0.99). Fewer transfusions were also linked to increased dysfunction risk, with an 11% reduction in risk per unit transfused (*p* = 0.030; OR = 0.89; 95% CI: 0.79–0.99). Additionally, higher body mass index emerged as a significant risk factor, with a 5% increase in dysfunction risk per body mass index (BMI) point (*p* < 0.001; OR = 1.05; 95% CI: 1.01–1.07). Preexisting renal dysfunction remained an important predictor, doubling the risk of DLGR (*p* = 0.006; OR = 2.03; 95% CI: 1.02–3.35).

**TABLE 4:** Multivariate logistic regression for risk factors of liver graft dysfunction.

Variables	Wald test	df	*p*	OR	95% CI	
Recipient						
Age	5.68	1	0.017	0.98	0.97	0.99
No. of transfusions	4.71	1	0.030	0.89	0.79	0.99
BMI	15.21	1	< 0.001	1.05	1.01	1.07
Renal dysfunction	7.69	1	0.006	2.03	1.02	3.35
Donor						
Age	12.46	1	< 0.001	1.02	1.01	1.03
Sex	6.80	1	0.009	1.69	1.14	2.50
Sodium	7.25	1	0.007	1.01	1.01	1.02
Graft						
Warm ischemia time	7.95	1	0.005	1.07	1.02	1.12
Cold ischemia time	3.91	1	0.048	1.002	1.001	1.003

*Note: p* < 0.05 was considered statistically significant.

Abbreviations: 95% CI: 95% Confidence interval; df: Degree of freedom; OR: Odds ratio.

Among donor variables, older age was directly associated with increased graft dysfunction risk, with a 2% increase per year (*p* < 0.001; OR = 1.02; 95% CI: 1.01–1.03). Male sex was also a significant predictor, with a 69% increase in the chance of DLGR (*p* = 0.009; OR = 1.69; 95% CI: 1.14–2.50). Higher serum sodium levels in the donor correlated positively with the outcome (*p* = 0.007; OR = 1.01; 95% CI: 1.01–1.02), indicating that electrolyte imbalances may impair graft quality. Warm ischemia time increased the likelihood of dysfunction by 7% per additional time unit (*p* = 0.005; OR = 1.07; 95% CI: 1.02–1.12), while cold ischemia time also showed a significant, though smaller, effect (*p* = 0.048; OR = 1.002; 95% CI: 1.001–1.003).

## 4 Discussion

The present study identified nine factors independently associated with DLGR, including recipient‐related variables (age, number of transfusions, body mass index, and renal dysfunction), donor‐related variables (age, serum sodium levels, and sex), and graft‐related variables (warm and cold ischemia times). The association between recipient age and DLGR is consistent with the findings of Etayo et al. (2023), who reported a higher prevalence of dysfunction among younger recipients, suggesting that age may be a relevant prognostic marker during the first year after transplantation. Furthermore, the multivariate regression analysis showed that recipients with DLGR were more likely to receive organs from older male donors with hypernatremia and prolonged warm and cold ischemia times.

The predominance of male sex among both donors and recipients observed in this study corroborates the findings of Etayo et al. (2023), who reported 75.5% of their sample as male, with a mean age of 54.8 years. The primary etiology for transplantation was cirrhosis in 86.7% of cases, and 67.4% of patients presented with associated comorbidities—in the present study, the main comorbidity was renal dysfunction. The literature also highlights other factors associated with graft dysfunction, including prior use of antiplatelet and/or anticoagulant agents, immunosuppression, and portal vein thrombosis (Etayo et al. 2023).

Although this study found a statistically significant association between hepatic encephalopathy and DLGR, no previous studies were identified to support this result, suggesting a possible spurious association. Regarding transfusions, the finding that recipients with DLGR received fewer blood transfusions appears to contrast with prior studies of early graft dysfunction (Lee et al. 2016). However, transfusion volume may reflect not only bleeding severity but also differences in perioperative hemodynamic management, blood conservation strategies, and the use of supportive therapies. In addition, because transfusion data were retrospectively obtained from medical records, this variable may represent an imperfect proxy for clinical severity. Survival‐related and residual confounding mechanisms may also have influenced this association. Therefore, this finding should be interpreted with caution and not as evidence of a protective effect of lower transfusion exposure. In this context, transfusion practices should be interpreted as part of a broader perioperative management strategy rather than as a direct proxy for graft injury alone.

Lee et al. (2016) reported that EAD is related to preoperative ventilatory status, donation after cardiac death (DCD) allografts, donor age, graft size, degree of steatosis, operative time, and transfusion volume. Furthermore, the severity of early dysfunction influences graft survival, with moderate EAD associated with poorer outcomes compared to no dysfunction (Wiemann et al. 2025). In the present sample, dysfunction was associated with fewer transfusions, higher BMI, and renal dysfunction.

The literature recognizes that intraoperative transfusion of more than 500 mL is indicated in the presence of tissue hypoxia or significant acute blood loss (Silva Junior et al. 2008). Excessive transfusion, in turn, is associated with increased rates of infectious complications and hospital mortality (26.3%), with complications occurring in 57.5% of cases, findings that are consistent with those of the present study.

Given the negative impact of allogeneic transfusions, alternative strategies have been explored. Pinto et al. (2019) discuss the use of autologous blood, particularly through intraoperative blood salvage (IBSA), to reduce transfusion‐related risks without compromising clinical outcomes. Although the study by Yanaral and Karaaslan (2022) demonstrated reduced blood loss with the use of a cell saver system, this benefit did not translate into significant clinical or transfusion‐related improvements. Yoon et al. (2022) further noted that transfusion of red blood cells and platelets was associated with increased mortality, whereas the use of prothrombin complex concentrate and fibrinogen concentrate did not significantly reduce intraoperative transfusion needs nor increase thrombotic events. Cell salvage and educational interventions were associated with reduced use of blood products.

Eghbal et al. (2019) emphasized that intraoperative blood loss during liver transplantation correlates with donor age, MELD score, warm ischemia time, and bilirubin levels. Although the correlation with the underlying liver disease was weak, it was still clinically relevant.

In the present study, recipients with DLGR had significantly higher BMI (26.44 vs. 25.61), consistent with the study by Alnagar et al. (2025), which showed that BMI > 25 kg/m^2^ is associated with a higher incidence of primary non‐function and early dysfunction. Additionally, recipients with hepatic dysfunction were twice as likely to present with renal dysfunction, a finding supported by Molnar et al. (2019), who demonstrated that recipients with preserved renal function prior to transplant had a lower risk of death with a functioning graft compared to those undergoing simultaneous liver‐kidney transplantation.

Donor‐related factors also contributed to DLGR. This study found a higher likelihood of male donors (OR = 1.69; 95% CI; *p* = 0.009), consistent with Grinspan et al. (2026), who reported 70% male donors. Literature suggests that men are more susceptible to liver disease due to greater exposure to risk factors such as alcohol use, up to three times higher than in women, which may compromise graft quality (Shi et al. 2025; Sarkoohi et al. 2025).

Elevated donor sodium levels were also more common among recipients with graft dysfunction, supporting the findings of Pollard et al. (2025), who identified sodium levels > 160 mmol/L as a risk factor for graft function. Murillo et al. (2026) also reported a mean donor sodium concentration higher than 155 mEq/L, similar to that observed in the present study. Furthermore, this study also identified prolonged warm ischemia time as significantly associated with graft dysfunction. These findings are supported by Ito et al. (2020) and Coffey et al. (2017), who linked prolonged warm ischemia to ischemia–reperfusion injury and early graft dysfunction.

This study presents several limitations that must be acknowledged. The retrospective nature of the study may have introduced biases related to incomplete or inaccurate medical record documentation, potentially affecting data quality. Additionally, the study was conducted in a single center, which may limit the generalizability of the findings. The complex triad of donor‐recipient‐graft variables includes elements beyond the transplant team's control, and undocumented factors may have influenced clinical decisions. Furthermore, the final sample size was smaller than originally estimated, reducing statistical power from 80% to 73%. This limitation may have impaired the detection of weaker associations, especially for variables with small effect sizes, and should be considered when interpreting nonsignificant results.

The retrospective case–control design also entails potential selection and sampling biases, since participants were not randomly allocated and group classification was based on outcomes identified in medical records. In addition, the inclusion of only complete and internally consistent records may have introduced selection related to data availability and record quality. Although cases and controls were drawn from the same source population and classified using predefined criteria, these limitations should be considered when interpreting the findings.

Although causal inference methods may provide important advantages in observational studies when the objective is to estimate the effect of a specific exposure under explicit causal assumptions, this was not the main purpose of the present study. Our objective was to identify factors associated with DLGR and to support the clinical and conceptual development of a nursing diagnosis. Therefore, logistic regression was used as an appropriate method for adjusted association analysis in a retrospective case–control study with a binary outcome. Future studies may benefit from causal inference approaches, particularly if focused on specific modifiable exposures and guided by prespecified causal models.

## 5 Conclusion

This study identified nine factors significantly associated with an increased risk of DLGR, demonstrating that dysfunctional liver graft response is a complex and multifactorial phenomenon influenced by recipient, donor, and intraoperative factors. Among recipients, higher body mass index, renal dysfunction, reduced transfusion support, and younger age were associated with increased risk. Among donors, older age, male sex, elevated serum sodium levels, and prolonged warm and cold ischemia times were associated with a greater likelihood of DLGR.

## 6 Relevance for Clinical Practice

The identification of independent predictors of DLGR has direct implications for clinical protocols and nursing practice in liver transplantation. From a policy perspective, the findings support the inclusion of nursing‐sensitive risk factors in frameworks for organ allocation, donor evaluation, and recipient monitoring. Standardizing the use of the proposed RDLGR diagnosis can strengthen care planning, interdisciplinary communication, and documentation of transplant‐related complications.

In clinical practice, these results emphasize the need for vigilant nursing assessment of younger recipients and of those with renal dysfunction, elevated BMI, and limited transfusion support. In addition, intraoperative and postoperative care protocols should address modifiable risk factors such as prolonged warm and cold ischemia times and donor electrolyte imbalances. Overall, the implementation of targeted nursing interventions guided by evidence‐based risk profiling may improve graft survival and patient outcomes in liver transplantation programs.

## Author Contributions

**Layana de Paula Cavalcante:** conceptualization, investigation, writing – original draft, methodology, validation, formal analysis, writing – review and editing. **João Cruz Neto:** writing – original draft, writing – review and editing, methodology, validation, formal analysis. **Maria Beatriz Freire Alves:** writing – review and editing, writing – original draft, investigation, methodology. **Francisca Alessandra Peixoto da Cunha:** writing – review and editing, writing – original draft, investigation, methodology. **Ana Clara de Brito Gomes:** writing – review and editing, writing – original draft, investigation, methodology. **Reinaldo Gutierrez Barreiro:** writing – review and editing, writing – original draft, investigation, methodology. **Viviane Martins da Silva:** supervision, conceptualization, methodology, writing – original draft, writing – review and editing. **Marcos Venícios de Oliveira Lopes:** supervision, conceptualization, methodology, writing – original draft, writing – review and editing.

## Funding

This research received specific grant from Conselho Nacional de Desenvolvimento Científico e Tecnológico—CNPQ. Processes: No. 310378/2023‐0 and 313 495/2021‐1.

## Ethics Statement

Fieldwork permissions were secured through the Hospital Universitário Walter Cantídio Research Ethics Committee (permit number: 5.208.291).

## Consent

The authors confirm that informed consent was obtained from the patient(s) for participation in this study and for the publication of relevant clinical information and images. Identifying details have been anonymized to protect patient privacy.

## Conflicts of Interest

The authors declare no conflicts of interest.

## Supporting information


**Data S1:** STROBE checklist.

## Data Availability

The data that support the findings of this study are available on request from the corresponding author. The data are not publicly available due to privacy or ethical restrictions.
